# Colorectal Cancer-Associated Microbiome Patterns and Signatures

**DOI:** 10.3389/fgene.2021.787176

**Published:** 2021-12-22

**Authors:** Lan Zhao, William C. Cho, Mark R. Nicolls

**Affiliations:** ^1^ Department of Medicine, Stanford University School of Medicine, Stanford, CA, United States; ^2^ VA Palo Alto Health Care System, Palo Alto, CA, United States; ^3^ Department of Clinical Oncology, Queen Elizabeth Hospital, Hong Kong, China

**Keywords:** gut microbiome, colorectal cancer, dysbiosis, biclustering, patient-microbe interactions, oral-related pathogens

## Abstract

The gut microbiome is dynamic and shaped by diet, age, geography, and environment. The disruption of normal gut microbiota (dysbiosis) is closely related to colorectal cancer (CRC) risk and progression. To better identify and characterize CRC-associated dysbiosis, we collected six independent cohorts with matched normal pairs (when available) for comparison and exploration of the microbiota and their interactions with the host. Comparing the microbial community compositions between cancerous and adjacent noncancerous tissues, we found that more microbes were depleted than enriched in tumors. Despite taxonomic variations among cohorts, consistent depletion of normal microbiota (members of *Clostridia* and *Bacteroidia*) and significant enrichment of oral-originated pathogens (such as *Fusobacterium nucleatum* and *Parvimonas micra*) *were* observed in CRC compared to normal tissues. Sets of hub and hub-connecting microbes were subsequently identified to infer microbe-microbe interaction networks in CRC. Furthermore, biclustering was used for identifying coherent patterns between patients and microbes. Two patient-microbe interaction patterns, named P0 and P1, can be consistently identified among the investigated six CRC cohorts. Characterization of the microbial community composition of the two patterns revealed that patients in P0 and P1 differed significantly in microbial alpha and beta diversity, and CRC‐associated microbiota changes consist of continuous populations of widespread taxa rather than discrete enterotypes. In contrast to the P0, the patients in P1 have reduced microbial alpha diversity compared to the adjacent normal tissues, and P1 possesses more oral-related pathogens than P0 and controls. Collectively, our study investigated the CRC-associated microbiome changes, and identified reproducible microbial signatures across multiple independent cohorts. More importantly, we revealed that the CRC heterogeneity can be partially attributed to the variety and compositional differences of microbes and their interactions to humans.

## 1 Introduction

Humans are made up of trillions of cells, which work together to carry out essential functions required for life. Besides human cells, there are many microorganisms living in and on our bodies, which are collectively called human microbiota (Micah et al., 2007). The term microbiome describes either the collective genomes of the microbiota, or the microorganisms themselves (Micah et al., 2007; Knight et al., 2017). The skin, mouth, and gastrointestinal tract all harbor different types of microorganisms as revealed by the Human Microbiome Project (HMP) (Micah et al., 2007). The majority of microbes reside in the gut, making it an attractive target for microbiome research. Diet, antibiotics, age, and environmental conditions have been shown to affect the composition of the gut microbiome (Claesson et al., 2012; Caesar et al., 2015; Langdon et al., 2016). For instance, a high-fat diet reduces the level of *Akkermansia muciniphila* and *Lactobacillus*, which are both beneficial for a healthy metabolic state (Caesar et al., 2015). The microbiota of older people (>65 years) displays greater inter-person variation, and lower diversity levels than that of younger adults (Claesson et al., 2012). Although the gut microbiome changes over time and can be affected by various factors, 60% of an individual’s microbiota remains stable for years or even decades, suggesting that microbial signatures might be useful for clinical evaluation of human diseases (Faith et al., 2013; Levy et al., 2020).

The interactions between human cells and gut microbes play important roles in human health and disease. Recent evidence showed that the gut microbiota is involved in the regulation of various human physiological processes including metabolic functions and immune systems (Wang et al., 2017; Li et al., 2021). On the other hand, dysbiosis (imbalance of microbiota) has been shown to be associated with a wide range of diseases (Wang et al., 2017) including inflammatory bowel disease (IBD), obesity, mental illnesses, and colorectal cancer (CRC). CRC is a growing public health problem worldwide. Dysbiosis is recognized as an important player in CRC initiation and progression (Kostic et al., 2013; Zackular et al., 2013; Wang et al., 2017; Cheng et al., 2020). For example, Zackular et al. (2013) found that changes in the gut microbiome directly contributed to tumorigenesis in mice. Pathogens such as *Fusobacterium nucleatum (F. nucleatum)* and *Bacteroides fragilis (B. fragilis)* were overabundant during disease progression from adenomas to CRC (Kostic et al., 2013; Cheng et al., 2020).

CRC is characterized with high heterogeneity and variability in molecular characters and clinical outcomes (Guinney et al., 2015). Four consensus subtypes (CMS1-CMS4) of CRC were defined by the Colorectal Cancer Subtyping Consortium (CRCSC) (Guinney et al., 2015). CMS1 patients have strong immune system activation; CMS2 tumors displayed epithelial differentiation; CMS3 is a genomically stable subtype with metabolic dysregulation; and CMS4 malignancies have the worst clinical outcomes, stromal invasion, and angiogenesis. CRC microbiota heterogeneity was previously investigated by researchers such as in (Flemer et al., 2017; Purcell et al., 2017). Purcell et al. (2017) identified CMS subtype specific microbial profiles from a cohort of 34 CRC patients, such as *F. nucleatum* was elevated in CMS1, and *Prevotella* species were enriched in CMS2. Flemer et al. (2017) stratified 59 CRC patients into 6 clusters: a Pathogen cluster, a *Prevotella* cluster, two clusters of *Bacteroidetes* and *Firmicutes*, respectively. In addition, Arumugam et al. (2011) performed multidimensional cluster analysis identified 3 distinct clusters of the human gut microbiome (designated as enterotypes), and the patterns were reproducible in other two cohorts. The enterotypes are mostly driven by closely related microbial species with similar taxonomy. Specifically, the enterotype 1 is enriched in *Bacteroides*, enterotype 2 is abundant with *Prevotella*, and the enterotype 3 is mostly a *Ruminococcus* rich group. The clusters identified from the last two studies resemble each other, suggesting that microbial composition changes in CRC patients may form small sets of discrete states.

The 16S ribosomal RNA (rRNA) gene is present and highly conserved among bacteria, which contains nine hypervariable regions (V1-V9) suitable for bacterial identifications. 16S rRNA gene amplicon sequencing is cost-effective and has been essential in identifying bacterial species in clinical samples (Burns et al., 2015; Gao et al., 2015). For example, Gao et al. (2015) used 16S rRNA gene V3 region to investigate microbiota changes between tumor and matched normal samples. They found that *Proteobacteria* phyla was under-represented, whereas *Firmicutes* phyla and *Fusobacteria* genus were over-represented in CRC. Burns et al. (2015) found an elevated abundance of *Providencia* in the tumor microenvironment by sequencing the V5-V6 regions of the 16S rRNA gene. CRC microbial compositions identified from different studies share similarities and differences, suggesting a meta-investigation of multiple cohorts is needed to identify robust microbial signatures for CRC.

Clustering is an unsupervised classification method to uncover the structures and patterns in data. K-means and hierarchical clustering are the two commonly used algorithms to partition either features or samples into different groups based on their similarities (Chang et al., 2014). Biclustering allows simultaneous clustering of both features and samples in order to identify coherent patterns from both dimensions (Madeira and Oliveira, 2004; Zhao et al., 2018). BackSPIN (Zeisel et al., 2015), a biclustering algorithm on single cells that iteratively splits both cells and genes, until no further splitting is needed. Like the generally sparse single cell data, the microbe-sample count matrices generated from microbial profiling studies are very sparse with many zero values, thus we did the attempt of using BackSPIN biclustering on microbial matrices to reveal potential CRC-microbe-interaction patterns. Meanwhile, previous studies on uncovering CRC microbiota heterogeneity were mostly conducted on faecal samples, which is generally considered to be representative for the distal part of the large intestine. Since faecal samples provide an incomplete and biased representation of gut microbiome, the analysis of mucosal/tissue is more directly related to the microbiota involvement in CRC physiopathology (Villéger et al., 2018).

Therefore, our study is to apply a meta-investigation of multiple independent CRC cohorts with matched tumor/normal tissue pairs (when available), not only to determine the consistently altered microbial species in CRC patients, but also to identify robust patient-microbe interaction patterns. More importantly, we revealed the CRC’s heterogeneity at the microbiome level, and found that only a subset of the CRC patients were identified to have significant microbial changes compared to normal controls.

## 2 Materials and Methods

### 2.1 Data Collection

We collected six independent CRC datasets, considering in total 353 patients and their matched normal mucosal samples (when available)’ 16S rRNA amplicon sequencing (16S) data (Table 1). All the samples included came from untreated patient tissues. More specifically, Kostic dataset (Kostic et al., 2012) has microbial sequencing of 95 CRC patients and matched normal controls, which contains the largest number of samples included in the study. 67, 65, and 44 tumor-normal pairs were subjected to 16S sequencing by Burns et al. (2015), Gao et al. (2017), Hale et al. (2018), respectively. Although most of the sequenced samples from Zeller dataset (Zeller et al., 2014) came from fecal samples, there were 48 tumor-normal mucosal pairs which were added into our study. And lastly, the included Purcell dataset (Purcell et al., 2017) contains 34 tumor samples without matched normal control subjects. Beside the Kostic dataset, which was based on 454 pyrosequencing, the remaining datasets were all generated by the Illumina MiSeq paired-end platform (Table 1).

**TABLE 1:** Size and characteristics of the CRC 16S datasets used in the study.

		Kostic	Hale	Gao	Zeller	Burns	Purcell
Datasets information	Source	Vall d’Hebron University Hospital	Mayo Clinic	Shanghai Tenth People’s Hospital	University Hospital Heidelberg	University of Minnesota	University of Otago
	16S Regions	V3-V5	V3-V5	V4	V4	V5-V6	V3-V4
	Technology	454 sequencing	Illumina MiSeq	Illumina MiSeq	Illumina MiSeq	Illumina MiSeq	Illumina MiSeq
	Reads	∼1,000 bp	2 × 300 bp	2 × 250 bp	2 × 250 bp	2 × 250 bp	2 × 250 bp
	Accession	SRP000383	PRJNA445346	PRJNA383606	PRJEB6070	PRJNA284355	PRJNA404030
Patients	Tumor	95	67	65	48	44	34
	P0	52	33	32	24	20	19
	P1	43	34	33	24	24	15
	P1%	45.3%	50.7%	50.8%	50.0%	54.5%	44.1%
	Average Age (P0)	NA	66.7	NA	63.7	63.6	70.6
	Average Age (P1)	NA	67.1	NA	66.1	66.2	78.2
	Normal	95	67	65	48	44	0
	Average Age (Normal)	NA	64.6	NA	64.9	64.9	NA
Microbes	Phylum	11	11	24	24	23	18
	Genus	205	212	478	495	562	318
	Species	420	415	318	286	269	231
	Lowly variable species	24	10	21	24	84	0
	P0-specific	226	216	162	141	85	149
	P1-specific	170	189	135	121	100	82
	P1-specific%	42.9%	46.7%	45.5%	46.2%	54.0%	35.5%

Patient’s clinical information, such as age, gender, and Body mass index (BMI) were downloaded (when available) from the corresponding publications (Kostic et al., 2012; Zeller et al., 2014; Burns et al., 2015; Gao et al., 2017; Purcell et al., 2017; Hale et al., 2018).

### 2.2 16S Data Processing

16S microbial profiles were obtained either by re-analysing the raw data, or by downloading the processed Amplicon sequence variant (ASV) tables, whichever is applicable. The ASV table for the Kostic dataset was obtained from the Microbiome Learning Repo (MLRepo) database (Vangay et al., 2019). Hale dataset’s ASV assignments were downloaded from the associated publication (Hale et al., 2018). For re-analysing the data, the raw sequences were downloaded from EBI-ENA database (accession number: PRJEB6070, PRJNA284355, PRJNA404030, and PRJNA383606), followed by processing the reads through the DADA2 (1.14.0) pipeline (Callahan et al., 2016). Specifically, we used DADA2 with the standard filtering parameters: maxEE = (2, 2), truncQ = 2, rm. phix = TRUE, and trimmed the potential adapter and primer sequences. The denoised forward and reverse reads were then merged, and chimeric sequences were removed. The taxonomy was assigned using the Silva reference database (version 132), and species level classifications based on exact matching between ASVs.

ASV count table, the taxonomic assignments, and patient’s metadata were combined into a phyloseq object for each dataset for further processing and virtualization. Rare ASVs with prevalence less than 1% of samples were excluded. Microbial count data was normalized to median sequencing depth, transformed to relative abundances, and log2-transformed after adding a pseudocount of 1.

### 2.3 Statistical Analysis of Microbial Community Data

Alpha diversity is the diversity within a particular habitat, and was calculated using Shannon diversity index in the study. Microbial relative abundances were used to calculate Shannon diversity index for each sample in a dataset. Differences were evaluated using the pairwise Wilcoxon rank sum tests with Benjamini-Hochberg (BH) correction. A *p*-value < 0.05 was considered statistically significant.

Beta diversity is commonly used to measure similarities and differences between samples. Principal coordinates analysis (PCoA) was performed based on Bray-Curtis distance to estimate the beta diversity of microbial communities. A permutational multivariate analysis of variance (PERMANOVA) was then performed using the “adonis2” function from the vegan package (Dixon, 2003) to test for differences between different microbial communities. The analysis was based on Bray-Curtis dissimilarity with 999 permutations, and accounted for by the covariates/confounders such as age, gender, and BMI (when available). *p*-values of 0.05 or lower were considered to be statistically significant.

Microbial differential abundance analysis (adjusted for patients’ clinical factors for subtype comparison) was performed using DEseq2 with the Phyloseq package in R. Results were considered significant if the BH adjusted *p*-value was less than 0.05.

### 2.4 Biclustering

BackSPIN (Zeisel et al., 2015), a divisive biclustering method based on sorting points into neighborhoods (SPIN) (Tsafrir et al., 2005), can be seen as simultaneous clustering of rows and columns of a data matrix. BackSPIN can help to identify coherent patterns between microbes and samples from microbial abundance data.

A filtering process was performed for the 16S species-level dataset to exclude the microbes with standard deviation (SD) of less than 0.05. The data were then fed into the BackSPIN algorithm for biclustering analysis with default parameters. The depth of clustering (d = 4) is specified as levels of binary splits the BackSPIN will be attempted, and a maximum of 2^4^ clusters will be created for each analysis (Zeisel et al., 2015). The optimal cluster number was determined by the Gap statistics (Tibshirani et al., 2001). Microbe-sample biclusters determined by BackSPIN and Gap statistics were displayed by heatmap visualizations.

### 2.5 Construction of Microbial Interaction Networks

A microbial interaction network consists of a collection of hub microbes and their connected microbes. Hub microbes are predicted to act as potential biomarkers that are either positively or negatively interacting with their connected microbes. In our analysis, we employed a network-based approach using ARACNe algorithm (Margolin et al., 2006) to investigate the microbe-microbe interactions in the development and progression of CRC. The differentially altered microbes (BH-adjusted *p* < 0.05) between groups (tumor vs. normal; P1 vs. P0) were considered as the hub microbes, and the set of microbes connected with a given hub microbe forms a sub-network.

Specifically, the microbe-microbe interaction network inference was performed in the RTN package (Fletcher et al., 2013), which executed in four major steps: 1) compute mutual information (MI) between a hub microbe and all potential connections with the remaining microbes; remove non-significant associations (Spearman’s coefficient correlation with BH corrected *p*-value less than 0.01) by RTN’s permutation calculations (nPermutations = 1,000); 2) remove unstable interactions (edges) by bootstrapping (the consensus fraction is 95%, and the number of bootstraps is 100); 3) apply the ARACNe algorithm; 4) build microbial interaction networks that are centered on hub microbes, and network visualization in the RedeR package (Castro et al., 2012).

Smaller networks with fewer than 5 hub microbes or edges were visualized by plotting heatmaps using the pheatmap package (with the default parameter settings) (Kolde, 2012). And the cutoff of removing non-significant associations among microbes was set as 0.05 (Spearman’s coefficient correlation with BH corrected).

### 2.6 Association Analysis Between Microbes and Patient Clinical Factors

Statistical tests such as the chi-squared test and the Mann-Whitney U-test were performed to test the patient grouping information with various clinical variables (such as age, gender, and BMI). BH corrected *p*-values of 0.05 or lower were considered to be statistically significant.

## 3 Results

### 3.1 A Survey of Microbial Composition Changes in Different CRC Cohorts

Six independent CRC 16S sequencing datasets, namely Kostic, Hale, Gao, Zeller, Burns, and Purcell were collected and analysed in our study. Datasets and patient cohorts’ characteristics were provided in Table 1. We used one percent of all samples in a dataset as the prevalence threshold to eliminate singleton and rare amplicon sequence variants (ASVs) for each cohort. ASVs were then collapsed to different taxonomic levels (Kingdom, Phylum, Genus, and Species) for further investigation. Few members of *Euryarchaeota* and *Thaumarchaeota* phyla derived from the Kingdom of Archaea have been identified from the Zeller, Burns, and Kostic datasets, and the remaining microbes all belonged to Bacterial Phylums.

**FIGURE 1:**
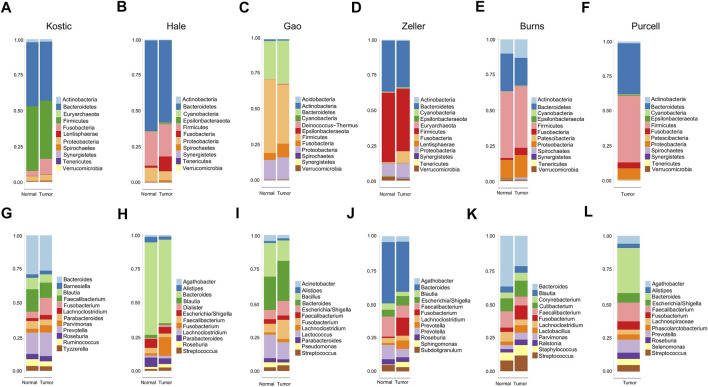
Relative abundance of top 12 microbial taxa from CRC patients and adjacent normal tissues. Taxa composition bar plots illustrate the relative microbial abundance (%) at the phylum level **(A–F)** and the genus level **(G–L)**. CRC patient cohorts include Kostic (a and g), Hale **(B, H)**, Gao **(C, I)**, Zeller **(D, J)**, Burns **(E, K)**, and Purcell **(F, L)**.

11 to 24 unique phyla have been identified from each of these six datasets (Table 1; Supplementary Table S1). *Firmicutes* and *Bacteroidetes* were the two dominant phyla, ranging from 63.6 to 85.2% in CRC gut microbiota. The other major phyla include *Proteobacteria, Fusobacteria*, and *Actinobacteria* (Figures 1A–F; Supplementary Table S1). Differential analysis indicated that increased proportions of *Fusobacteria* and/or *Epsilonbacteraeota* have been observed from the tumors compared to adjacent noncancerous tissues among most of the cohorts surveyed in the study (adjust *p* < 0.05; Supplementary Table S2). Phylum level composition variations have been observed among different CRC cohorts. For example, in cancerous tissues, *Bacteroidetes* with a percentage range from 58.0% in Hale to 19.8% in Burns dataset. And the percentages of *Fusobacteria* in the tumor group are 11.1% (Kostic), 5.1% (Burns), 8.4% (Zeller), 10.1% (Hale), 9.5% (Gao), and 4.3% (Purcell) (Figures 1A–F; Supplementary Table S1).

When we assessed the differences at the level of genera, we found a diverse category of bacteria genera ranging from 205 (Kostic) to 562 (Burns) (Table 1). *Bacteroides, Prevotella, Fusobacterium, Faecalibacterium, Blautia*, and *Lachnoclostridium* are among the most abundant genera (Figures 1G–L). Microbial composition variations among CRC cohorts have also been observed at the genus level (Figures 1G–L; Supplementary Table S1). For instance, in the top 12 genera, the percentages of *Faecalibacterium* ranging from 2.6% (Hale) to 14.0% (Purcell); and the percentage of the *Bacillus* genera was 15.1% in Gao compared to 1.0% in Hale dataset (Supplementary Table S1). Phylum and genus levels microbial composition variations suggested that gut microbiota composition has cohort differences. Differential analysis between tumor and normal groups across cohorts indicated that the genera of *Faecalibacterium, Alistipes*, and Blautia have been enriched in normal tissues, and tumor-enrichment for *Fusobacterium, Selenomonas*, and *Campylobacter* have been commonly observed across CRC cohorts (adjust *p* < 0.05; Supplementary Table S2).

**FIGURE 2:**
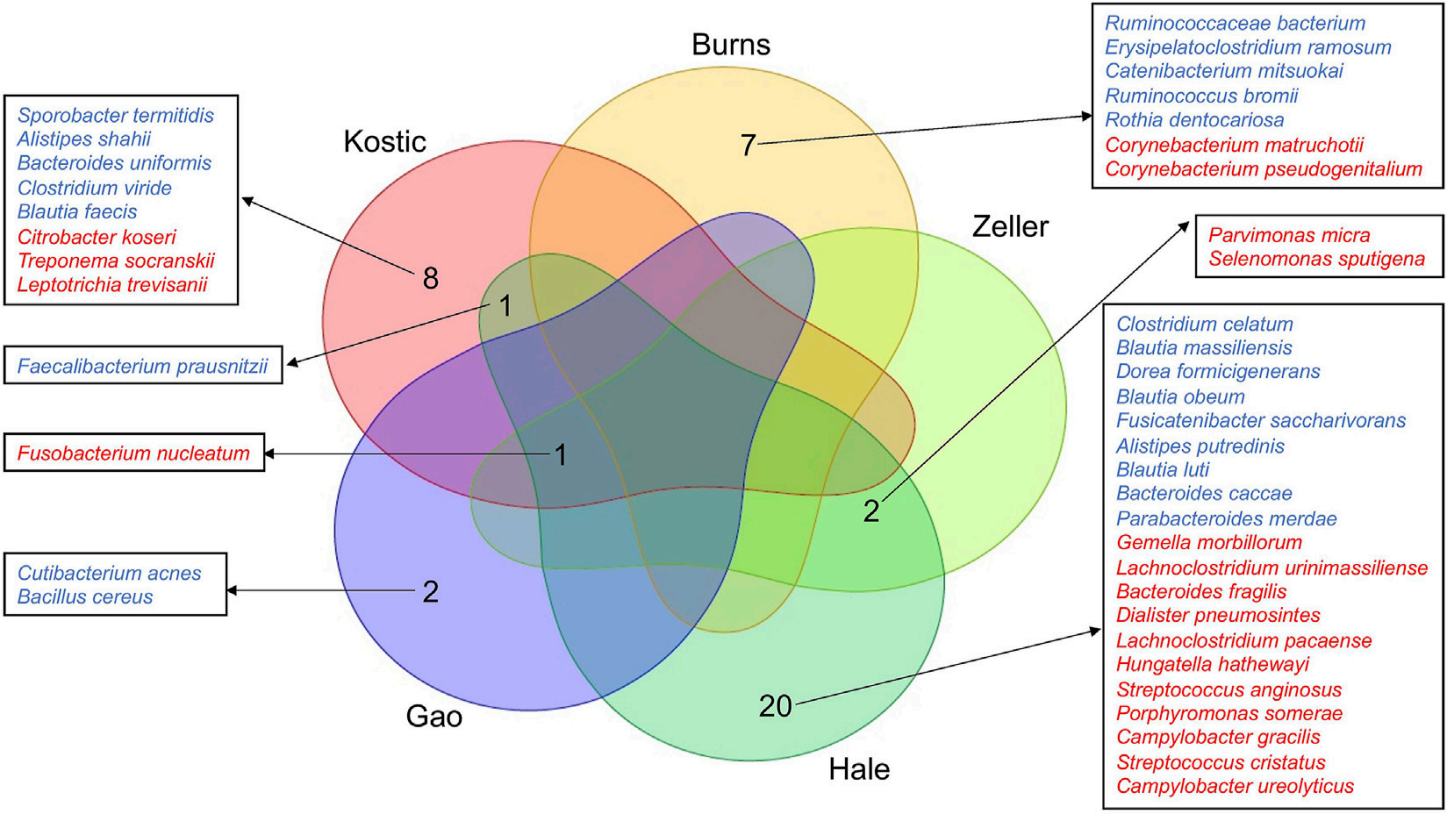
Differentially altered microbial species between tumor and normal groups, and their overlap relationships across the five CRC cohorts. The significantly differentially enriched and depleted microbial species between tumor and normal groups (adjust *p* < 0.05) were compared for their overlap relationships across the five CRC cohorts (Kostic, Hale, Gao, Zeller, and Burns). The number of overlap species across the cohorts were shown by a Venn diagram. Species names were shown around the Venn diagram. Tumor-enriched species were marked in red, and tumor-depleted species in blue.

A total of 420, 415, 318, 286, 269, and 231 microbial species have been detected from Kostic, Hale, Gao, Zeller, Burns, and Purcell datasets, respectively (Table 1; Supplementary Table S3). Among them, 62 common species have been detected in these six datasets, and majority of them came from the order of *Bacteroidales* and *Clostridiales* (Supplementary Table S4). The significantly differentially enriched and depleted species between tumors and normals (adjust *p* < 0.05) and their overlap relationships across the datasets were illustrated in Figure 2. Multiple species have been found to be uniquely enriched/depleted among different CRC cohorts (Figure 2), suggesting that the microbial composition variations were common at the species level. Four species, namely *F. nucleatum* (4 datasets), *F. prausnitzii* (2 datasets), *P. micra* (2 datasets), and *S. sputigena* (2 datasets) were detected to be significantly altered in more than one dataset (Figure 2). Specifically, aside from the Burns dataset, *F. nucleatum* has been found to be enriched in tumors compared to adjacent normal tissues in the Kostic, Hale, Gao, and Zeller datasets. Tumor depletion of *F. prausnitzii* has been observed from the Kostic and Hale cohorts (Figure 2).

We didn’t observe any significant microbial diversity (Shannon alpha-diversity index) differences between the tumor and normal samples at any of the six cohorts (Supplementary Figure S1). Bray-Curtis distance was computed to measure the dissimilarity between tumor and normal microbial compositions (beta diversity), and principal coordinates analysis (PCoA) revealed highly significant differences between the two groups on the phylum, genus, and species levels (PERMANOVA, adjust *p* < 0.05; Supplementary Figure S2). Taken together, these findings suggested that gut microbiota composition has both cohort differences and similarities; most importantly, significant microbial community composition differences, but not the microbial alpha diversity difference between cancerous and adjacent noncancerous tissues have been detected in different CRC cohorts.

### 3.2 Differential Microbial Interaction Networks in Kostic and Hale Cohorts

Like the genes in a genome, microorganisms in the gut microbiota interact. To examine the underlying microbe-microbe interaction network in CRC, we inferred a differential microbial interaction network (DMIN) using ARACNe algorithm (Margolin et al., 2006) for each CRC cohort investigated. Differentially altered microbes between tumor and normal were selected as the hub microbes, and the set of hub-connecting microbes by a given hub microbe forms a sub-network. The microbial interaction units (sub-networks) were thus composed of the hub microbes and their connected microbes. The degree distribution of the network was defined as the number of microbes connected with the hub microbes, which was used to represent the importance of the hub microbes (the more the bigger the importance). Kostic and Hale cohorts were first selected based on the criteria: more than 5 hub microbes or edges, to investigate the potential microbe-microbe interactions.

**FIGURE 3:**
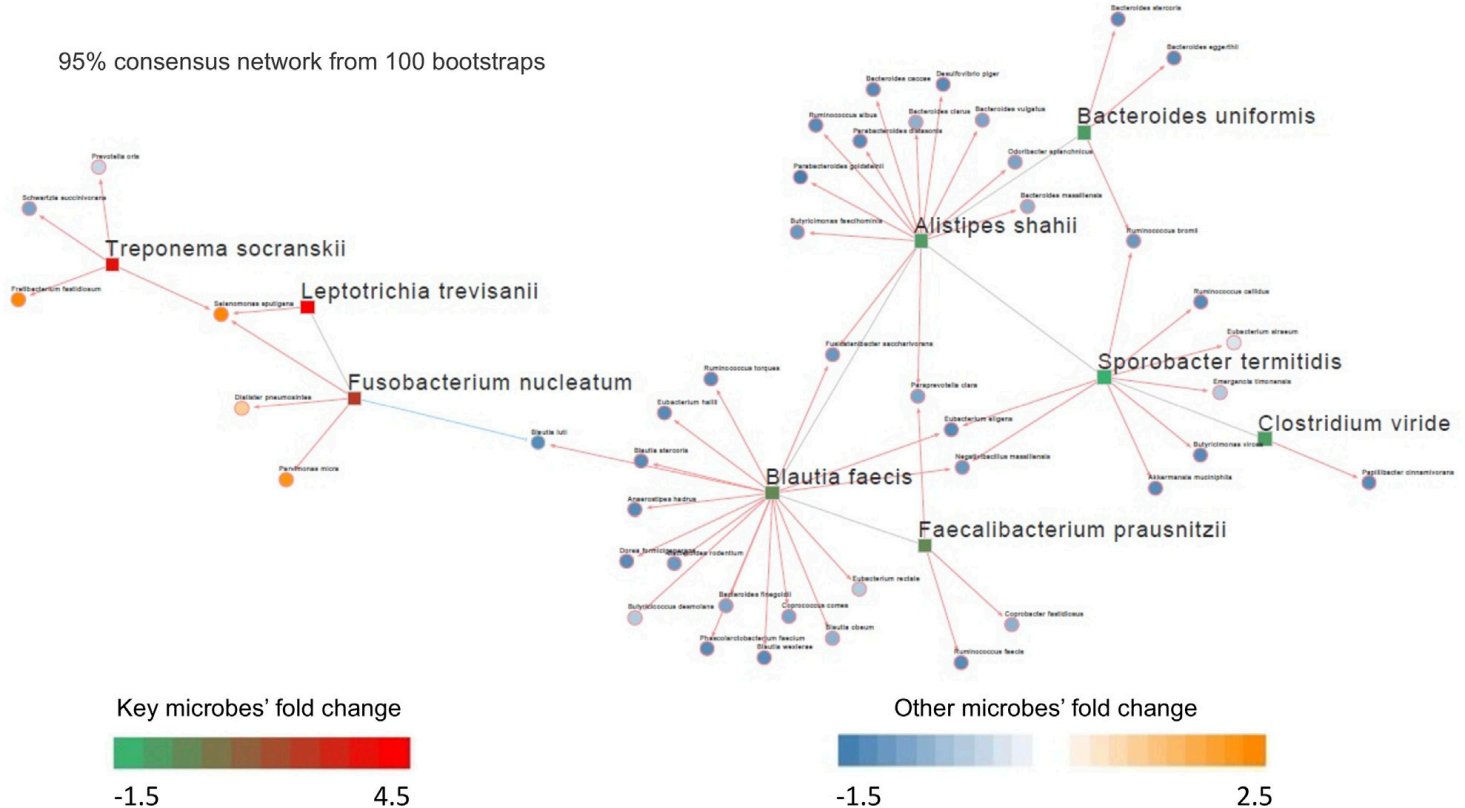
Differential microbial interaction networks in the Kostic cohort. A directed graph displaying differential microbial species between tumor and normal groups (hub microbes) in the Kostic cohort (shown in rectangles; green: depleted in tumor, red: enriched in tumor). Microbes predicted to interacting with the hub microbes were shown based on their differential level between tumor and normal groups (shown in circles; blue: depleted in tumor, orange: enriched in tumor). The connections among microbes were depicted in red (induction) or blue (repression) based on the associations of the hub microbes and their related microbes.

Out of the 10 microbes differentially enriched/depleted between tumor and normal samples in the Kostic dataset (adjust *p* < 0.05; Supplementary Table S2), 9 (*S. termitidis, A. shahii, B. uniformis, C. viride, F. prausnitzii, B. faecis, F. nucleatum, T. socranskii,* and *L. trevisanii*) were predicted to be the hub microbes (Figure 3). Among them, 3 were enriched (shown in red rectangles), and the remaining 6 (shown in green rectangles) were depleted in tumors. Of the 3 up-altered species in tumors, two were derived from the order of *Fusobacteriales*, and the remaining 1 (*T. socranskii*) is a pathogen which can cause diseases in humans. The 6 depleted microbes in tumors all come from the phyla of *Firmicutes* and *Bacteroidetes*, most prominently in the order of *Clostridiales*. Microbes predicted to be associated with the hub microbes are shown in orange (highly enriched in tumor) and blue (depleted in tumor). More microbes were depleted than enriched (48 vs 7) in tumors. 6 (*F. nucleatum, T. socranskii, F. fastidiosum, S. sputigena, D. pneumosintes*, and *P. micra*) of the 7 enriched microbes were oral pathogens (Chen et al., 2010) (Table 2), indicating the role of oral microbiome on the tumorigenesis of CRC. 59 connections (edges), which were weighted by Spearman’s correlation coefficients among microbes were added to the network (Figure 3). *B. faecis, A. shahii, and S. termitidis* were the top 3 largest hub microbes which were associated with 17, 14, and 10 microbe’s abundances, respectively. Almost all the interactions between hub microbes and their connected microbes were positive (cooperative), except for a negative (competitive) relationship between *F. nucleatum* and *B. luti*. *B. luti* is a beneficial bacteria, which was depleted in tumors, its relationship with *F. nucleatum* needs further investigation.

**TABLE 2:** The prevalence of the 15 oral-related microbes in the six datasets.

Species	Kostic (%)	Hale (%)	Gao (%)	Zeller (%)	Burns (%)	Purcell (%)
*Fusobacterium nucleatum*	70.5	49.1	60.0	75.0	22.7	61.8
*Treponema socranskii*	21.1	4.3	9.2	8.3	9.1	8.8
*Fretibacterium fastidiosum*	11.6	4.8	9.2	16.7	0.0	14.7
*Selenomonas sputigena*	40.0	10.4	26.2	35.4	4.5	44.1
*Dialister pneumosintes*	50.5	35.2	46.2	50.0	18.2	14.7
*Parvimonas micra*	82.1	27.0	64.6	66.7	43.2	38.2
*Solobacterium moorei*	6.3	13.0	0.0	47.9	2.3	5.9
*Dialister pneumosintes*	50.5	35.2	46.2	50.0	18.2	14.7
*Peptoanaerobacter stomatis*	2.1	18.7	69.2	2.1	15.9	29.4
*Selenomonas infelix*	0.0	4.3	12.3	10.4	2.3	17.6
*Fretibacterium fastidiosum*	11.6	4.8	9.2	16.7	0.0	14.7
*Treponema socranskii*	21.1	4.3	9.2	8.3	9.1	8.8
*Filifactor alocis*	4.2	3.5	6.2	8.3	4.5	0.0
*Porphyromonas endodontalis*	15.8	0.0	13.8	6.3	0.0	0.0
*Campylobacter gracilis*	8.4	9.1	3.1	20.8	4.5	14.7

Based on the 24 differentially abundant bacteria species in the Hale dataset (adjust *p* < 0.05; Supplementary Table S2), 22 were selected to build the DMIN which consisted of 22 hub microbes with 143 edges (Supplementary Figure S3). There were 2 overlapped hub microbes (*F. nucleatum* and *F. prausnitzii*) between Hale and Kostic dataset. Hub microbes’ connection size range from 1 (for *S. sputigena*) to 12 (for *F. prausnitzii*). Similar to our previous results, oral pathogens (such as *C. gracilis, S. sputigena*, and *P. micra*) were enriched in tumors; most of the depleted species were belonging to the order of *Clostridiales*; and hub microbes were mostly positively interacted with their connected microbes, indicating potential symbiotic relationships between them.

### 3.3 Differential Microbial Correlation Networks in Other CRC Cohorts

**FIGURE 4:**
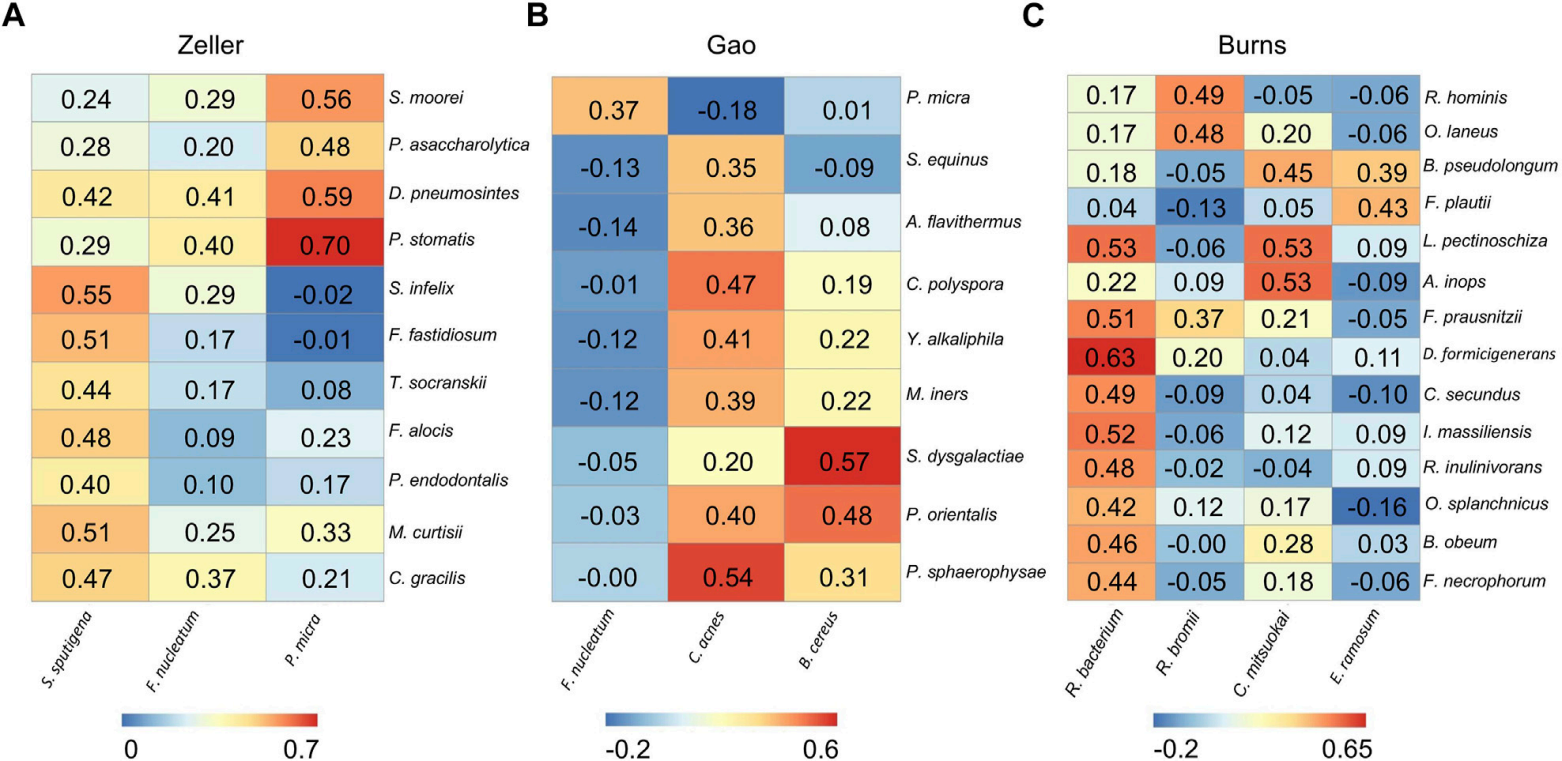
Differential microbial correlation networks in other cohorts. Heatmaps display a number of statistically significant Spearman’s coefficient correlations (BH corrected *p* < 0.05) between hub microbes and their related microbes in Zeller **(A)**, Gao **(B)**, and Burns **(C)** cohorts. Numbers in the heatmaps represent the correlation coefficient values. Each column represents a hub microbe, with the species names shown at the bottom. Each row represents a hub-connecting microbe, with the species names shown on the right.

The remaining three CRC cohorts (Zeller, Gao, and Burns) which have tumor-normal pairs were employed separately to infer differential microbial correlation networks (DMCNs), as they have less than 5 hub microbes or edges. DMCNs were constructed according to the Spearman’s correlation coefficients between hub microbes and their connected microbes (adjust *p* < 0.05) and were visualized by heatmaps (Figures 4A–C).

From the Zeller dataset, 3 hub microbes (*S. sputigena, F. nucleatum, and P. micra*) were all enriched in tumors, and were significantly positively correlated with a total of 11 microbes’ relative abundance (Figure 4A). 9 of the 11 microbes have an oral origin (*S. moorei, D. pneumosintes, P. stomatis, S. infelix, F. fastidiosum, T. socranskii, F. alocis, P. endodontalis, and C. gracilis*) (Chen et al., 2010) (Table 2), and the remaining 1 bacteria (*M. curtisii*) is associated with *Bacterial vaginosis* (BV).

3 hub microbes were also identified from the Gao dataset. Consistent with previous reports, *F. nucleatum* was enriched in tumors compared to normal controls. Correlation heatmap analysis of the relationship between hub microbes and their connected microbes showed that *F. nucleatum* was positively interacted with *P. micra*, although the correlation was not strong (0.367) (Figure 4B). The remaining two hub microbes (*C. acnes, and B. cereus*) were all depleted in tumors. *C. acnes* is an opportunistic pathogen, and the stains of *B. cereus* are widespread in our living environment (Majed et al., 2016).

There are two microbial species from the genus of *Corynebacterium* that were enriched in tumors, but not selected as the hub microbes in the Burns cohort. Among the 4 hub microbes (all depleted in tumors, which are belonging to the normal gut microbiota) in the Burns cohort, *R. bacterium* was the most significant microbe to be interacted with other microbes (Figure 4C). For instance, *R. bacterium* positively interacts with *D. formicigenerans* with the Spearman’s correlation coefficients as 0.63.

In summary, the DMCNs inferred from Zeller, Gao, and Burns were similar to those obtained earlier, that is, pathogens (like *F. nucleatum*) were mostly cooperative associated with their targeted microbes, the majority of them have an oral-origin and were highly enriched in the tumor site; whereas beneficial microbes (members of *Clostridiales*) were depleted in tumors, and positively interacted with each other.

### 3.4 Biclustering Identifies Two Microbial Subtypes of CRC

We have examined the microbial composition changes and investigated the potential interactions between hub microbes and their connected microbes in different CRC cohorts. As CRC is heterogeneous at the molecular level (Guinney et al., 2015), we next asked the question if CRC is heterogeneous at the microbial level.

**FIGURE 5:**
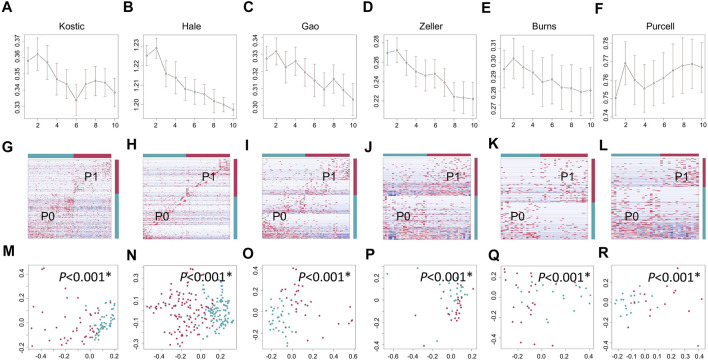
Biclustering identified two microbial subtypes in CRC. Gap statistics (and their standard deviations) per cluster number (1–10), which reach peaks at k = 2 for Kostic **(A)**, Hale **(B)**, Gao **(C)**, Zeller **(D)**, Burns **(E)**, and Purcell **(F)**. Species level abundance heatmaps display biclustering results for Kostic **(G)**, Hale **(H)**, Gao **(I)**, Zeller **(J)**, Burns **(K)**, and Purcell **(L)**. Two patient-microbe interaction patterns (P0 and P1) were consistently identified across the CRC cohorts investigated. Red represents the P1 group, and blue for the P0 group. Each row represents the abundance for each species, with the species’ grouping information indicated by the colored bar located on the right. Each column represents the abundance for each patient, with the patient subtyping information indicated by the colored bar located on the top. The PCoA based on Bray-Curtis dissimilarity was used to estimate the beta diversity of microbial communities for Kostic **(M)**, Hale **(N)**, Gao **(O)**, Zeller **(P)**, Burns **(Q)**, and Purcell **(R)**. Each point represents a patient. P1 patients were in red, and P0 patients in blue. PERMANOVA was used to test for differences between P1 and P0. *p*-values of 0.05 or lower were considered to be statistically significant, and marked with star.

**FIGURE 6:**
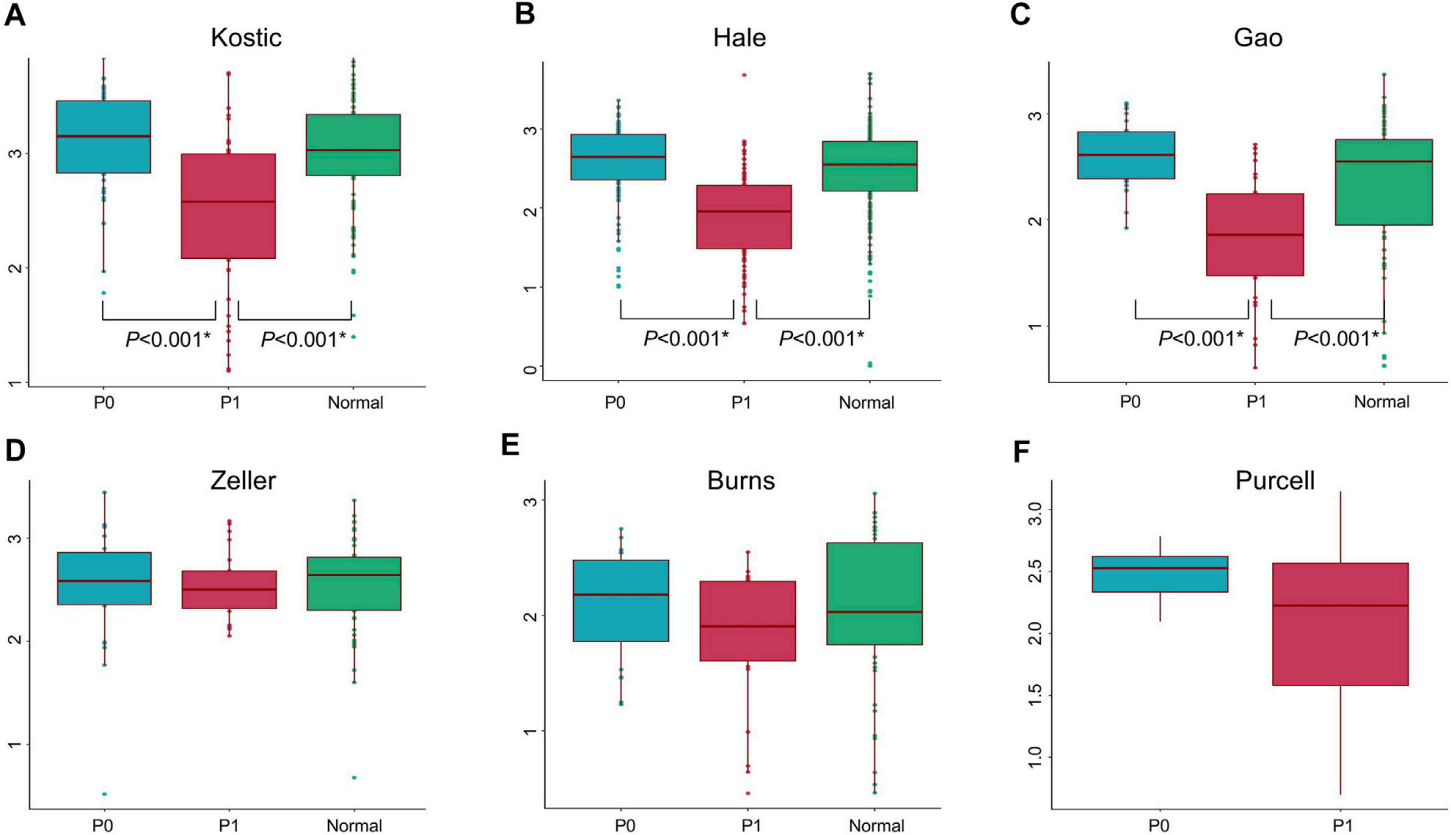
Microbial alpha diversity comparisons in different groups and cohorts. Shannon’s diversity index (in y-axis) was used to measure alpha diversity for Kostic **(A)**, Hale **(B)**, Gao **(C)**, Zeller **(D)**, Burns **(E)**, and Purcell **(F)**. P0 groups were colored blue, P1 were colored red, and Normal were colored green. Alpha diversity differences between groups were evaluated using the pairwise Wilcoxon rank sum tests with Benjamini-Hochberg (BH) correction. Only statistically significant *p*-values (less than 0.05) were shown below the boxplots, and marked with star.

Lowly variable species (SD < 0.05) for each cohort (Table 1) were filtered out before analysing through the BackSPIN, a biclustering approach to identify CRC subtypes that were co-perturbed across a subset of the microbes. The gap statistic compares the total within intra-cluster variability, and was used to determine the optimal number of clusters for each cohort. The previous PCoA analysis indicated that the microbial communities of a tumor and the corresponding normal samples from a given patient were more similar to each other than the tumors or paired normal samples from unrelated patients (Supplementary Figure S2), which is similar with the hierarchical clustering results obtained from Kostic et al. (2012). Thus, only tumor samples from each cohort were included in the biclustering analysis, and normal-adjacent samples were only involved when comparing the alpha diversity between patient subtypes and nearby intact tissues. Two CRC-microbe coherent patterns (P0 and P1) can be consistently identified from the six CRC cohorts, respectively (Figures 5A–F,G–L). Significant difference in the microbial alpha diversity were observed between the two subtypes in Kostic, Hale, and Gao cohorts (pairwise Wilcoxon test, adjust *p* < 0.05; Figures 6A–C); however, no significant pairwise microbial abundance differences were found between P1 and P0 subtypes in Zeller, Burns, and Purcell cohorts after multiple hypothesis correction (Figures 6D–F). More importantly, no statistically significant differences in microbial alpha diversity were observed between P0 patients and normal controls, whereas the alpha diversity in P1 patients were significantly decreased compared to P0 patients and non-tumor tissues (in Kostic, Hale, and Gao cohorts) (Figures 6A–C). PERMANOVA analysis of the Bray-Curtis distance (after adjusting for age, gender, and BMI effects when available) indicated that the P1 patients exhibited different beta microbial diversity than those of the P0 patients’ microbiomes (PERMANOVA, adjust *p* < 0.05; Figures 5M–R). Taken together, the PCoA on beta-diversity analysis revealed significantly distinct microbial compositions between the two subtypes in the six cohorts. In addition, the average microbial alpha diversity abundances in P1 patients were detected for less than that in P0 patients and normal controls in the Kostic, Hale, and Gao cohorts (Figures 6A–C).

### 3.5 Characterization of the CRC Microbial Subtypes

**FIGURE 7:**
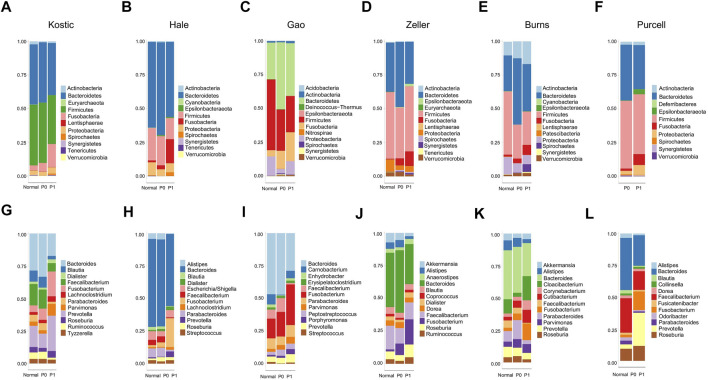
Relative abundance of top 12 microbial phyla/genera in different groups and cohorts. Taxa composition bar plots illustrate the relative microbial abundance (%) at the phylum level **(A–F)** and the genus level **(G–L)**. CRC patient cohorts include Kostic **(A, G)**, Hale **(B, H)**, Gao **(C, I)**, Zeller **(D, J)**, Burns **(E, K)**, and Purcell **(F, L)**.

An average of 45.1% of the total microbial species, ranging from 35.5% (Purcell) to 54.0% (Burns), were assigned into the P1 subtypes (Table 1); and the remaining species were belonging to each of P0 subtypes among the six cohorts, respectively. A total of 12 overlapped bacteria species, including 6 *Bacteroidales* and 6 *Clostridiales*, were present in P0 subtype across the six cohorts (Table 3). These species are part of normal gut flora, and are generally considered to be beneficial for gut health. For example, *B. uniformis, B. vulgatus*, and *F. prausnitzii* are among the most predominant commensal bacteria in the human intestine (Jansson et al., 2009). *R. bromii* within the order of *Clostridiales*, is responsible for the degradation of resistant starch (Ze et al., 2012). And *P. goldsteinii* possesses probiotic properties (Wu et al., 2019). 3 identical bacteria species were present in P1 subtype across the cohorts investigated (Table 3). Two of the three species were derived from the order of *Clostridiales*, and the other one came from the *Eggerthella* genus. *C. cadaveris* has been sporadically reported to be associated with human infection (Kiu et al., 2017). And *E. lenta* has been documented to induce bacteremia (Lee et al., 2014). The microbial taxa within each subtype were consistently diverse, composed of the five major phyla *Firmicutes, Bacteroidetes, Proteobacteria, Fusobacteria*, and *Actinobacteria* as observed earlier (Figures 1A–F, 7A–F). Each cohort individually had a list of dominated taxa, and in general, *Bacteroides, Prevotella, Fusobacterium*, and *Faecalibacterium* were among the most abundant genera within each subtype across the six CRC cohorts (Figures 7G–L), which further support that the gut microbiomes consist of continuous populations of widespread taxa rather than discrete enterotypes.

**TABLE 3:** Recurring subtype-specific microbial species in the six datasets.

Subtype	Kingdom	Phylum	Class	Order	Family	Genus	Species
P0	*Bacteria*	*Bacteroidetes*	*Bacteroidia*	*Bacteroidales*	*Bacteroidaceae*	*Bacteroides*	*Bacteroides coprocola*
P0	*Bacteria*	*Bacteroidetes*	*Bacteroidia*	*Bacteroidales*	*Bacteroidaceae*	*Bacteroides*	*Bacteroides uniformis*
P0	*Bacteria*	*Bacteroidetes*	*Bacteroidia*	*Bacteroidales*	*Bacteroidaceae*	*Bacteroides*	*Bacteroides vulgatus*
P0	*Bacteria*	*Bacteroidetes*	*Bacteroidia*	*Bacteroidales*	*Marinifilaceae*	*Odoribacter*	*Odoribacter splanchnicus*
P0	*Bacteria*	*Bacteroidetes*	*Bacteroidia*	*Bacteroidales*	*Tannerellaceae*	*Parabacteroides*	*Parabacteroides merdae*
P0	*Bacteria*	*Bacteroidetes*	*Bacteroidia*	*Bacteroidales*	*Tannerellaceae*	*Parabacteroides*	*Parabacteroides goldsteinii*
P0	*Bacteria*	*Firmicutes*	*Clostridia*	*Clostridiales*	*Ruminococcaceae*	*Faecalibacterium*	*Faecalibacterium prausnitzii*
P0	*Bacteria*	*Firmicutes*	*Clostridia*	*Clostridiales*	*Lachnospiraceae*	*Blautia*	*Blautia obeum*
P0	*Bacteria*	*Firmicutes*	*Clostridia*	*Clostridiales*	*Lachnospiraceae*	*Dorea*	*Dorea longicatena*
P0	*Bacteria*	*Firmicutes*	*Clostridia*	*Clostridiales*	*Lachnospiraceae*	*Dorea*	*Dorea formicigenerans*
P0	*Bacteria*	*Firmicutes*	*Clostridia*	*Clostridiales*	*Lachnospiraceae*	*Fusicatenibacter*	*Fusicatenibacter saccharivorans*
P0	*Bacteria*	*Firmicutes*	*Clostridia*	*Clostridiales*	*Ruminococcaceae*	*Ruminococcus_2*	*Ruminococcus bromii*
P1	*Bacteria*	*Actinobacteria*	*Coriobacteriia*	*Coriobacteriales*	*Eggerthellaceae*	*Eggerthella*	*Eggerthella lenta*
P1	*Bacteria*	*Firmicutes*	*Clostridia*	*Clostridiales*	*Clostridiaceae_1*	*Clostridium_sensu_stricto_2*	*Clostridium cadaveris*
P1	*Bacteria*	*Firmicutes*	*Clostridia*	*Clostridiales*	*Lachnospiraceae*	*Tyzzerella_4*	*Tyzzerella nexilis*

We next investigated the associations between CRC subtype assignments with patients’ clinical factors such as age, gender, and body mass index (BMI), as these factors were considered to influence the microbial community compositions (Claesson et al., 2012; Gao et al., 2018). The percentages of patients in subtype P1 were found to be 45.3% (Kostic), 50.7% (Hale), 50.7% (Gao), 50.0% (Zeller), 54.5% (Burns), and 44.1% (Purcell) (Table 1). Gao dataset doesn’t have any patient clinical information, and was excluded from this analysis. The remaining five datasets all have age and gender information. Besides that, BMI values for Zeller and Hale datasets were also available. Age and/or gender were not significantly associated with the subtype labels in the Kostic, Zeller, and Hale datasets, indicating that the microbial subtypes were independent from these factors. However, more older patients were enriched in P1, as identified from Burns and Purcell cohorts (Mann-Whitney U-test, BH adjust *p* < 0.05). In addition, P1 patients were more likely to be females, as observed solely in the Burns dataset (chi-squared test, BH adjust *p* < 0.05). We speculated the associations (between subtype labels with age and gender) were not convincing, as Burns and Purcell cohorts have relatively fewer patients (15–24) than others. Furthermore, from Zeller and Hale cohorts, we saw P1 patients have significantly higher BMI than P0 patients (Mann-Whitney U-test, BH adjust *p* < 0.05). Given the incomplete information and limited sample size for subgroup analyses, the associations between microbial subtype labels with clinical factors such as age, gender, and BMI were not robust despite being significant at some levels. In other words, the two CRC microbial subtypes were considered to be independent and not influenced by the patients’ metadata.

### 3.6 CRC Subtype-specific Microbes

We next performed the differential abundance analysis for identifying microbial members associated with CRC subtype status. To facilitate comparisons across datasets, we adjusted for age, gender, and BMI (when available) effects when performing the analysis, and Gao dataset was excluded from the analysis for the same reason. Zeller, Burns, and Purcell datasets were also not processed further as there were no differential abundance microbes between P0 and P1 subtypes after adjusting the confounding factors.

**FIGURE 8:**
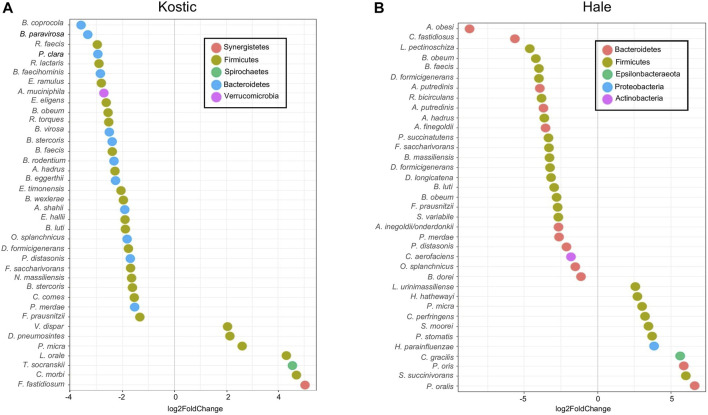
Differentially altered microbial species between P1 and P0 in Kostic and Hale cohorts. The significantly differentially enriched and depleted microbial species between P1 and P0 (adjust *p* < 0.05) with their log2 fold changes (in x-axis) were visualized by scatterplots for Kostic **(A)** and Hale **(B)** cohorts. Species names were shown on the left axis of the scatterplots. Each point on a scatterplot represents a microbial species, shown as a small colored circle, indicating the particular phylum they derived from.

Kostic and Hale datasets were used for subtype-specific microbes identification. Significant differences in the abundance of a number of taxa including *Prevotella, Clostridiales* and *Bacteroidales* were seen between P0 and P1 subtypes (adjust *p* < 0.05). More microbes were depleted than enriched in P1, and the majority of the depleted microbes in P1 were derived from the *Firmicutes* and *Bacteroidetes* phyla, most prominently in the order of *Clostridiales* (Figures 8A,B). Among the 38 bacteria being differentially abundant between P1 and P0 subtypes in the Kostic dataset, 7 oral-related species (*F. fastidiosum, C. morbi, T. socranskii, L. orale, P. micra, D. pneumosintes,* and *V. dispar*) were significantly enriched in P1 subtype (adjust *p* < 0.05; Figure 8A). 37 bacteria have been found to be differentially abundant between P0 and P1 subtypes in the Hale dataset (adjust *p* < 0.05). 11 of them were enriched, and the remaining 26 were depleted in P1 (Figure 8B). 7 out of the 11 enriched species (*P. micra, S. moorei, P. stomatis, H. parainfluenzae, C. gracilis, P. oris, and P. oralis*) were oral-related. *P. micra,* an oral-related pathogen which can cause a broad range of infections in humans (Carretero et al., 2016; Shinha and Caine, 2016), was enriched in P1 both from the Kostic and the Hale datasets. The relative abundance of *Clostridiales* and *Bacteroidales* in P0 subtype were higher than that in P1 subtype. Of the 10 overlapped species enriched in P0 (*B. faecis, B. obeum, B. luti, F. saccharivorans, P. distasonis, D. formicigenerans, O. splanchnicus, A. hadrus, F. prausnitzii, and P. merdae*) were identified both from the Kostic and Hale datasets, 7 were derived from the order of *Clostridiales*, and the remaining 3 came from the *Bacteroidales*. Most members of *Clostridiales* and *Bacteroidales* were found among the healthy gut microbiota, suggesting that P1 patients’ microbiota were more similar to controls than to the P0 patients.

## 4 Discussion

Gut microbiomes play important roles in the onset and progression of CRC. 16S rRNA gene amplicon sequencing is a cost-effective approach for microbiome studies. In this meta-investigation study, we systematically analyzed six independent 16S CRC cohorts in terms of their microbiota compositions. *Firmicutes, Bacteroidetes, Proteobacteria, Fusobacteria*, and *Actinobacteria* account for the majority of the gut microbiota. Although microbial composition variations have been observed among different CRC cohorts, tumor-specific enrichment of *F. phylum*, as well as depletion of *Clostridia* and *Bacteroidia* have been observed in CRC patients relative to normal controls. To explore this further, microbe-microbe and patient-microbe interaction networks were built to investigate the global and local patterns in the data. We found that hub microbes mostly positively interacted with their connected microbes. We also used BackSPIN, a biclustering approach to identify coherent CRC subtypes according to their abundance concordance in subtype-relevant microbes. BackSPIN can not only address the heterogeneity of CRC, but also identify microbes which are specific for each subtype. Through our analysis, we consistently identified two distinct CRC microbial subtypes across the cohorts investigated. One subtype, namely P1, showed decreased microbial diversity, lack of beneficial microbes, and was enriched for species associated with oral infections. However, the gut microbiota of the P0 patients resemble that from normal controls, indicating that only a subset of the cancer patients have suffered from intestinal dysbiosis; and dysbiosis accounts for partial CRC heterogeneity.

It was previously thought that gut microbiomes fall primarily into three discrete enterotypes: type 1 is enriched with *Bacteroides*, type 2 has less *Bacteroides* but *Prevotella* are predominant, and type 3 is a *Ruminococcus* rich group (Arumugam et al., 2011). The concept of enterotypes has been challenged recently (Knights et al., 2014; Gorvitovskaia et al., 2016), as gut microbial communities changing over time and presenting continuous gradients in compositions between *Bacteroides, Prevotella, Ruminococcus*, and many other taxa. Our study not only proved that gut microbiota composition has both cohort differences and similarities, but also further supports the evidence that gut microbiomes are spanning multiple co-occurring taxa which work together to interact with the host. A few hub microbes, including members of *Clostridiales* and *F. nucleatum*, were identified driving the compositions of the human gut microbial community. Members of *Clostridiales* are among the major constituents in the gut microbiome. In our study, we identified a broad range of interactions among *Clostridiales* in relation to other beneficial microbes in the tissue site. But the interactions are weaker and sensitive, and can be disrupted by pathogens, resulting in dysbiosis. Differential microbial interaction analysis between tumor and normal groups confirmed a number of correlations between *F. nucleatum* and other oral species, which are in agreement with previous results (Kolenbrander et al., 1989; Edwards et al., 2006). For instance, *F. nucleatum* has been tested to co-aggregate with a broader variety of oral bacteria (Kolenbrander et al., 1989), and the co-aggregation with *S. cristatus* to facilitate invasion of the later into host cells has been validated (Edwards et al., 2006). The Shedding, co-occurrence, and overgrowth of the oral cavity microbiomes in the digestive system not only implies that cancer patients are prone to microbial infection, but also provides opportunities to develop and use the specific probiotics and antibiotics to treat disease like CRC.

Gut microbes interact with the host in many ways, from nutrient uptake and immunity to chronic inflammation and carcinogenesis. Depletion of normal gut microbiota composition (members of *Clostridia*) as well as the presence of pathogens (like *F. nucleatum*) will disrupt the balance between microbe and host. Most members of *Clostridia* are typically anaerobic fermenters, known for their capacity for butyrate production. For example, *F. prausnitzii* is one of the main butyrate producing-bacteria in the human gut, and is reduced in abundance in many intestinal disorders (Lopez-Siles et al., 2017). *Roseburia spp.* of the family *Lachnospiraceae* are part of commensal bacteria, which produce butyrate to inhibit NF-κB activation and induce the maturation of the immune system (Tamanai-Shacoori et al., 2017). Butyrate is involved in a variety of metabolic and immune functions, and acts as a mediator for maintaining intestinal homeostasis. It is not only the preferred energy source for the colonocytes, but also has anti-inflammatory properties (Venegas et al., 2019). On the other hand, the presence of certain bacteria in the gut are harmful and can induce dysbiosis and tumorigenesis. For instance, since Kostic et al. (2012) established the association of oral pathogen *F. nucleatum* with CRC, more and more studies (Témoin et al., 2012; Yang et al., 2017; Sun et al., 2019) have been carried out to investigate the oncogenic mechanisms of *F. nucleatum* in CRC. The tumor-promoting role of *F. nucleatum* via activating toll-like receptor 4 (TLR4) signaling pathway has been observed in mice (Yang et al., 2017). Two *F. nucleatum* virulence factors: FadA and Fap2 were believed to create a pro-inflammatory microenvironment, which promote cancer development. FadA stimulates tumor cell proliferation (Témoin et al., 2012), and Fap2 can bind to immune cells causing immunosuppression (Sun et al., 2019). Besides the direct interactions with tumor and immune cells, toxins produced by microbes can not only damage the tissues, but also contribute to colon carcinogenesis. For example, toxins secreted by *C. difficile* can cause serious intestinal damage (Di Bella et al., 2016). Cyanotoxins produced by *Cyanobacteria* can affect multiple human organs, and play a role in colon cancer formation (Kubickova et al., 2019). And intestinal inflammation can be mediated by enterotoxigenic *Bacteroides fragilis* (ETBF) (Sears et al., 2014). Thus, the study of the microbiome-derived metabolome would be valuable.

## 5 Conclusion

In conclusion, gut microbes live in a constantly changing environment. The composition of microbial communities varies across different cohorts and populations. Taxonomically and functionally related microbes tend to co-exist. In CRC, microbiome shifts are dominated by the over-representation of a small number of oral-originated pathogens as well as the depletion of wide-ranging commensal intestinal bacteria. Two microbiome-based CRC subtypes have been identified, with significant differences in microbial compositions and abundance. Gut microbiomes contribute to CRC pathogenesis, and account for partial CRC heterogeneity.

## Data Availability

The original contributions presented in the study are included in the article/Supplementary Material, further inquiries can be directed to the corresponding authors.

## Supplementary Material

Supplementary Figure 1 Microbial alpha diversity at different taxonomy levels in tumor and normal groups. Shannon’s diversity index (in y-axis) was used to measure alpha diversity at the phylum level **(A–F)**, the genus level **(G–L)**, and the species level **(M–R)**. CRC patient cohorts include Kostic (a, g, and m), Hale **(B, H, N)**, Gao **(C, I, O)**, Zeller **(D, G, P)**, Burns **(E, K, Q)**, and Purcell **(F, L, R)**. *p*-values were not included in the boxplot as none of them were significant (Pairwise Wilcoxon rank sum tests with BH adjusted *p*-value < 0.05).

Supplementary Figure 2 Microbial beta diversity at different taxonomy levels in tumor and normal groups. The PCoA based on Bray-Curtis dissimilarity was used to estimate the beta diversity of microbial communities at the phylum level **(A–F)**, the genus level **(G–L)**, and the species level **(M–R)**. CRC patient cohorts include Kostic (a, g, and m), Hale **(B, H, N)**, Gao **(C, I, O)**, Zeller **(D, G, P)**, Burns **(E, K, Q)**, and Purcell **(F, L, R)**. Each point represents a sample. Tumor samples were in red, and normal samples in green. PERMANOVA was used to test for differences between tumor and normal groups. *p*-values of 0.05 or lower were considered to be statistically significant, and marked with star.

Supplementary Figure 3 Differential microbial interaction networks in the Hale cohort. A directed graph displaying differential microbial species between tumor and normal groups (hub microbes) in the Hale cohort (shown in rectangles; green: depleted in tumor, red: enriched in tumor). Microbes predicted to interacting with the hub microbes were shown based on their differential level between tumor and normal groups (shown in circles; blue: depleted in tumor, orange: enriched in tumor). The connections among microbes were depicted in red (induction) or blue (repression) based on the associations of the hub microbes and their related microbes.
