# Supplementary figures and images for: Colorectal Cancer-Associated Microbiome Patterns and Signatures
Source: Front Genet. 2021 Dec 22;12:787176. doi: 10.3389/fgene.2021.787176 (PMC8729777; doi:10.3389/fgene.2021.787176)

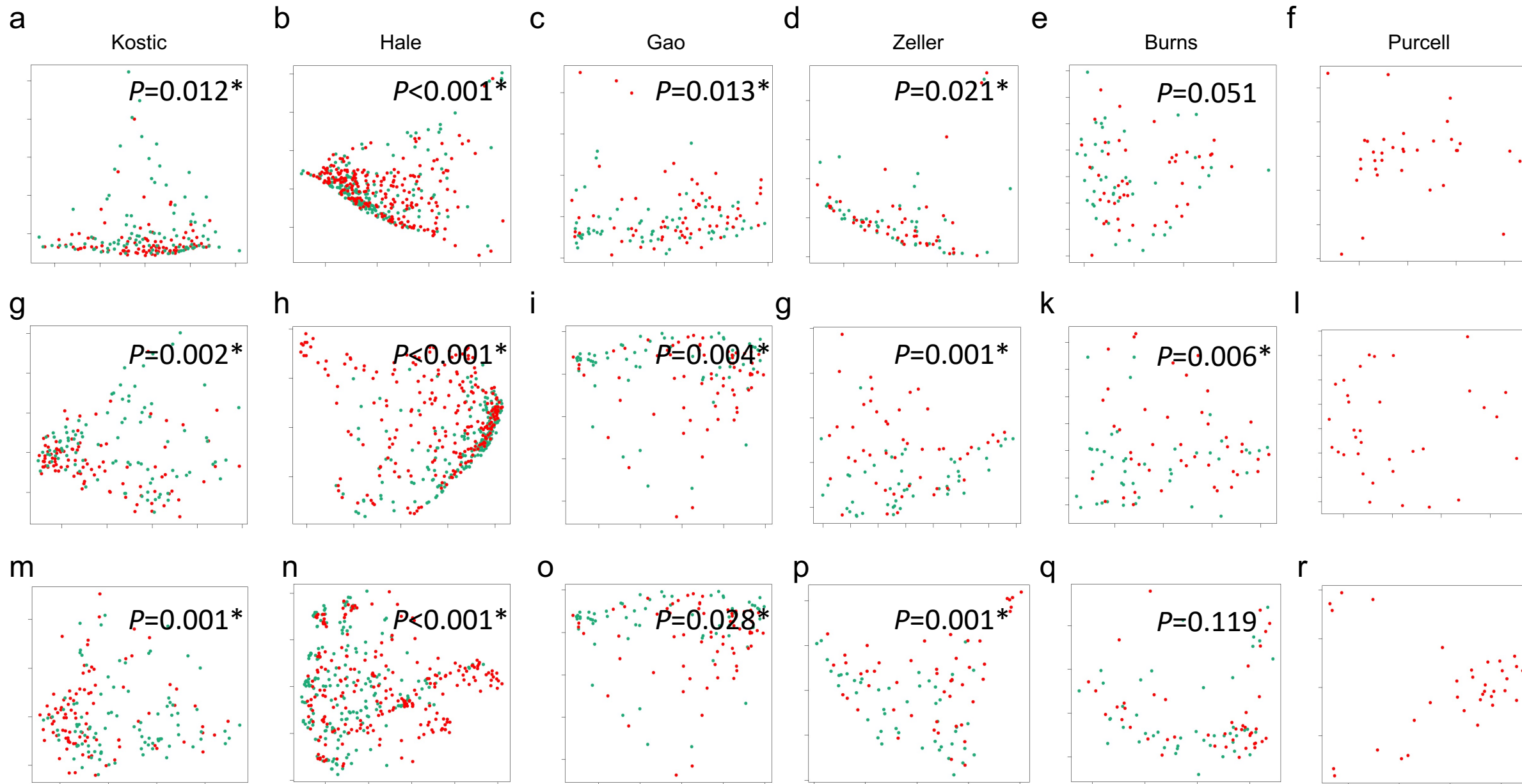

Supplement: Supplementary file 2 [file Image2.PDF]

95% consensus network from 100 bootstraps

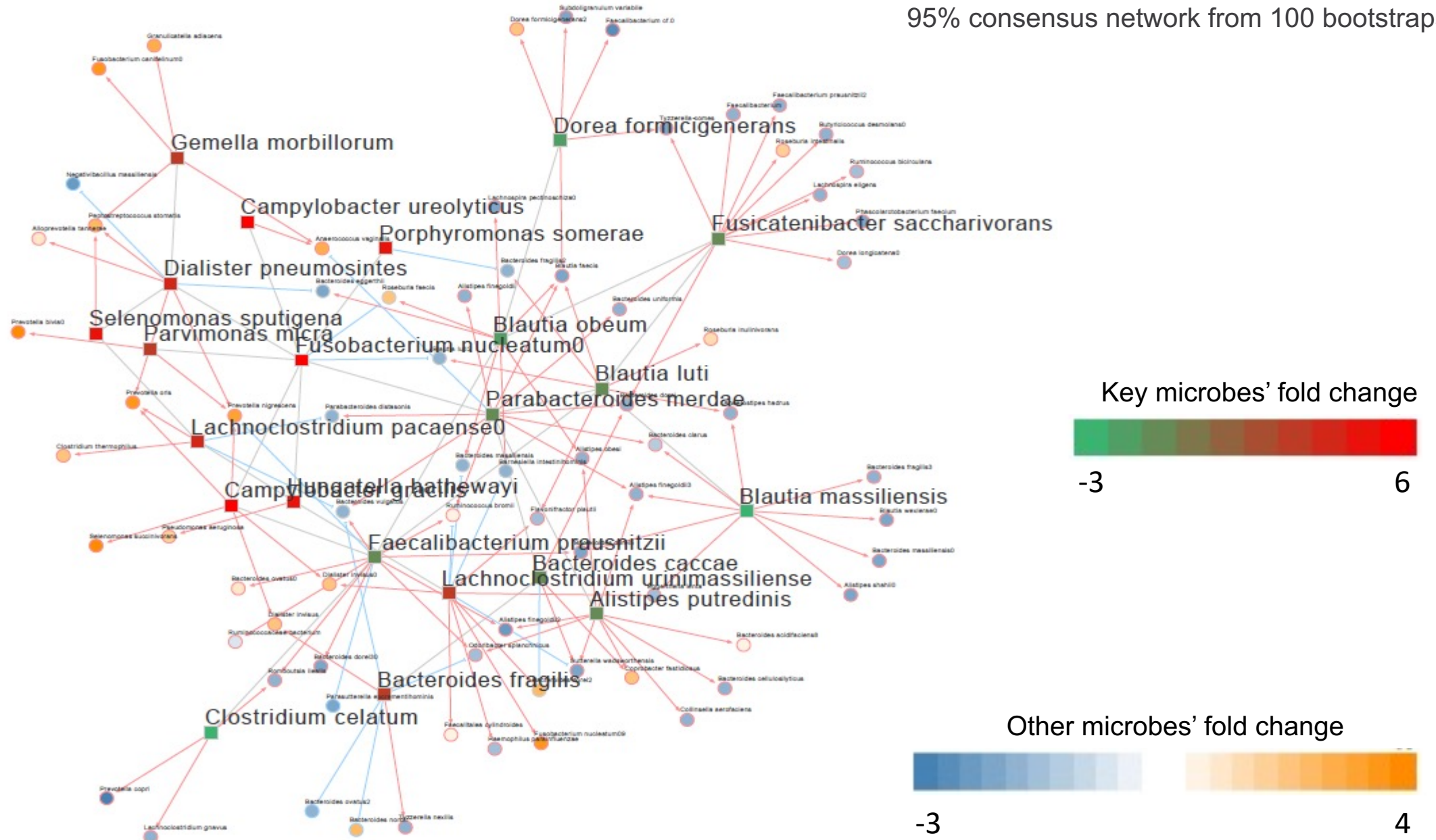

Supplement: Supplementary file 3 [file Image3.PDF]

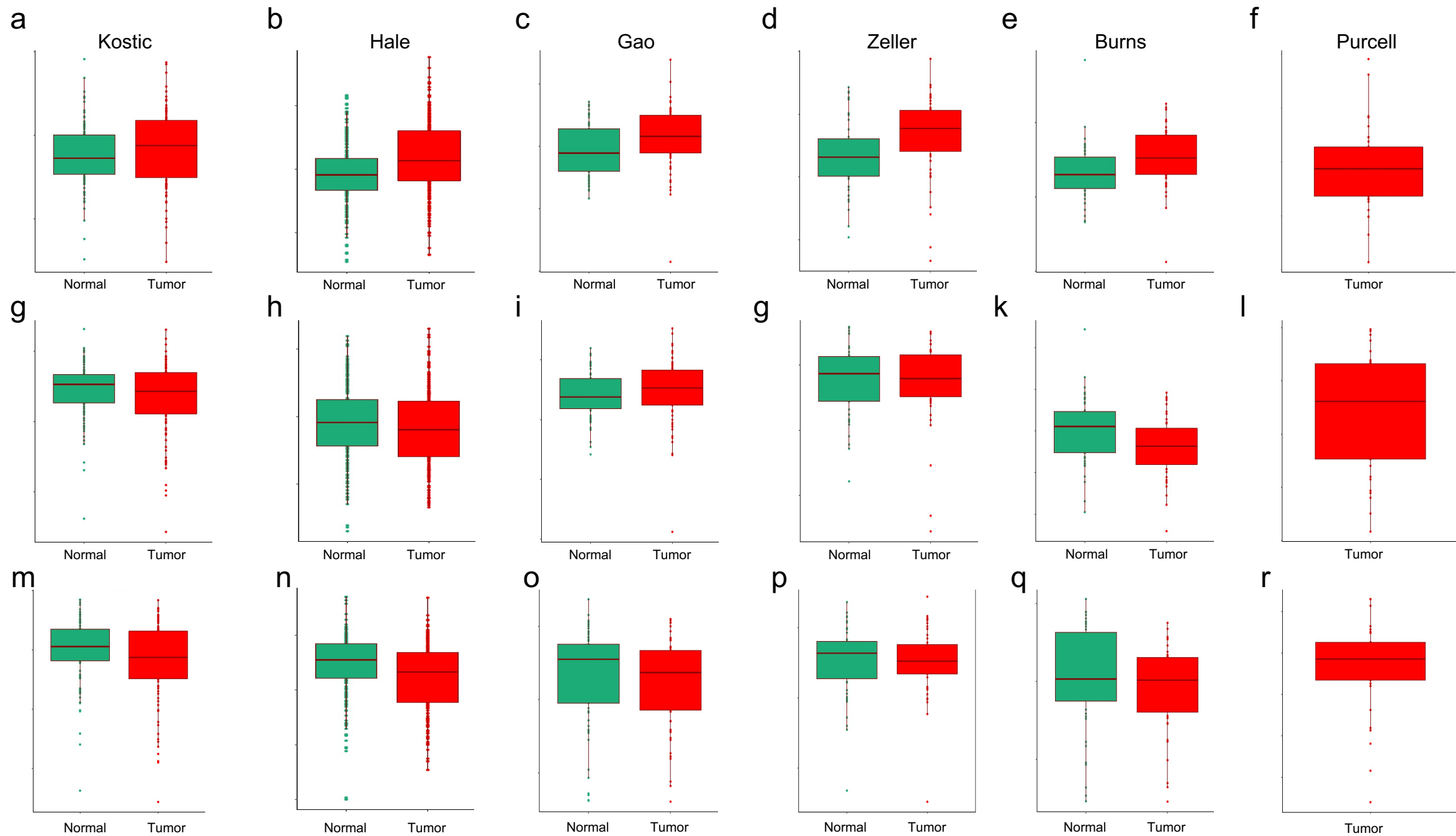

Supplement: Supplementary file 5 [file Image1.PDF]
